# Genetic variability of two populations of *Pseudoplatystoma reticulatum* from the Upper Paraguay River Basin

**DOI:** 10.1590/S1415-47572009005000075

**Published:** 2009-12-01

**Authors:** Marcia Matos de Abreu, Luiz Henrique Garcia Pereira, Vilma Barretto Vila, Fausto Foresti, Claudio Oliveira

**Affiliations:** 1 Departamento de Ciências Ambientais, Universidade do Estado de Mato Grosso, Cáceres, MTBrazil; 2 Departamento de Ciências Biológicas, Universidade do Estado de Mato Grosso, Cáceres, MTBrazil; 3 Departamento de Morfologia, Instituto de Biociências, Universidade Estadual Paulista Júlio de Mesquita Filho', Botucatu, SPBrazil

**Keywords:** fish, population genetics, microsatellite, homing, population structure

## Abstract

Catfishes of the genus *Pseudoplatystoma* are very important species due to both their high commercial value and their ecological role as voracious predators. They undertake lengthy migratory movements during their life-cycle, this including reproductive migration which occurs from October to December in the rainy season. In the present study, seven microsatellite loci were analyzed to access genetic variability in two samples of *P. reticulatum* from the Upper Paraguay Basin. The loci were highly polymorphic (mean = 7.28). According to all analysis, the two samples of *P. reticulatum* revealed pronounced genetic differentiation. *F*_st_ value was 0.2290, *R*_st_ value 0.1067 and AMOVA 22.90% (*F*_st_ ) and 10.67% (*R*_st_ ), all being highly significant (p < 0.001). The division of the fishes into two groups was confirmed by microsatellite multi-locus Bayesian assignment testing. The results obtained present evidence of genetic structuring in a *P. reticulatum* population.

Catfishes of the genus *Pseudoplatystoma* belong to the family Pimelodidae, the 93 species of which occurring in the major river basins of South America (Ferraris, 2007). They are migratory species of high commercial value, besides playing an important ecological role in the basins where they occur, due to their predatory behavior (Sato *et al.*, 1988; Miranda, 1997). The upriver migratory movement for reproduction usually occurs from October to December (Resende, 2003). Almost all the stocks of these giant catfishes had declined in the last years (Barthem and Goulding, 2007). The loss of biodiversity in aquatic environments is one among the most serious problems faced by countries all over the world (Moyle and Leidy, 1992), compromising the ecosystem functioning as a whole. The conservation of these ecosystems is vital for various economical sectors in many countries (Ehrlich and Ehrlich, 1992). According to Allan and Flecker (1993), several factors have been identified as causing the decline of fish diversity in several aquatic ecosystems, such as the introduction of exotic species, industrialization, urbanization, destruction of forests and riparian vegetation, pollution by pesticides and gold mining camps, and the construction of physical barriers for the generation of electricity.

The Pantanal area is located at the Upper Paraguay River Basin, with a drainage basin extending from the border between Brazil and Paraguay up to the limits of the Amazon River Basin, and covering about 140,000 km^2^ (Vila da Silva, 1995). In the Pantanal, fisheries constitute the second activity in economic importance. Furthermore, and apart from their ecological importance, fish resources are fundamental for subsistence, amateur, professional and sports fisheries, and the transaction of native crafts (Catella, 2003).

Microsatellite markers have been extensively used in studies on the genetics of fish populations. These markers have a co-dominant inheritance pattern, a high degree of polymorphism, and allow for easy analysis through experiments involving the polymerase chain reaction (PCR) (Wright and Bentzen, 1994; Triantafyllidis *et al.*, 2002; Salgueiro *et al.*, 2003; Barroso *et al.*, 2005; Mãkinen *et al.*, 2006). Studies using microsatellites have revealed pronounced genetic differences even among populations isolated by short geographic distances (Koskinen *et al.*, 2002). In this study, we analyzed two natural populations of *Pseudoplatystoma reticulatum* (“cachara”) collected in the Upper Paraguay River Basin, by using seven microsatellite loci originally developed for *P. corruscans* to access the genetic diversity of these samples. These data can be used as subsidies for setting up management policies for the development and conservation of these species.

A total of 52 adult specimens of *P. reticulatum* were caught by gill nets at two points on the Paraguay River Basin. 31 specimens were collected from the Paraguay River (PRP) (16° 04' 00" S 57° 41' 00" W) and 21 from the Jauru River (PRJ) (15° 51' 00" S 58° 27' 00" W). Fin clips were the source of nuclear DNA. These were collected from freshly caught fish and immediately preserved in 95% ethanol. After collection, the fishes were sold. For the extraction of genomic DNA, about 0.1 mg of tissue was incubated in 200 μL of 5% Chelex (Sigma®) at 65 °C overnight.

The *P. reticulatum* samples were screened for variation at each of the seven microsatellite loci. Five of these loci have already been described by Revaldaves *et al.* (2005) (Pcor01, Pcor05, Pcor08, Pcor10, and Pcor21), and two by Pereira *et al.* (2009) (Pcor23 and Pcor28). PCR amplification reactions were conducted in a thermocycler PTC-100 (MJ Research) with a final volume of 12.5 μL, consisting of about 10 ng of DNA, 0.25 μM of each primer, 0.2 mM of dNTP, 1.2 mM of MgCl_2_, 0.2 U of *Taq*-*Pht* DNA polymerase, 1X PCR buffer (50 mM KCl, 10 mM Tris-HCl, 0.1% Triton X-100, and 1.5 mM MgCl_2_) and water. We used the following PCR profile for the loci *Pcor01, Pcor02, Pcor05, Pcor08, Pcor21, Pcor23* and *Pcor28*: initial denaturation at 95 °C for 5 min, 30 cycles of 10 s at 95 °C, 15 s at an annealing temperature of 55 °C, 15 s at 72 °C and a final extension at 72 °C for 10 min. For the locus *Pcor10*, the PCR profile consisted of an initial denaturation at 95 °C for 5 min, 30 cycles of 30 s at 95 °C, 30 s at an annealing temperature of 48 °C, 30 s at 72 °C and a final extension at 72 °C for 10 min. Amplified products were resolved on 6% polyacrylamide gels stained with silver nitrate. Microsatellite alleles were identified by their size in base-pairs. Allele lengths were estimated by comparison with a 10 bp ladder (10 pb DNA Ladder - Invitrogen), using Kodak Digital Science 1D software.

Allelic count, expected and observed heterozygosity (*H*_E_, *H*_O_), inbreeding coefficient (*F*_IS_) and gene flow (*Nm* = 0.25(1-*F*_*ST*_)/*F*_*ST*_) were obtained with POPGEN 1.32 software (Yeh and Boyle, 1997). Allelic richness and Nei gene diversity were obtained with Fstat v2.9.3 software (Goudet, 2001). Deviation from Hardy-Weinberg equilibrium (HWE) was tested with the GENEPOP 3.3 package (Raymond and Rousset, 1995). MICRO-CHECKER 2.2.1 (van Oosterhout *et al.*, 2004) software was used to infer the most probable cause of HWE departures.

In order to investigate the genetic structure in samples of *P. reticulatum*, *F*_ST_ (Weir and Cockerham, 1984) indices, assuming the infinite allele model (IAM), and *R*_ST_ (Slatkin, 1995), which assumes the stepwise mutation model (SMM), were calculated for all loci using ARLEQUIN 3.11 (Excoffier *et al.*, 2005). Their significance estimates were based on 1,000 permutations. Analysis of molecular variance (AMOVA) (Excoffier *et al.*, 1992), incorporated in ARLEQUIN 3.11 (Excoffier *et al.*, 2005), was used to check the partition of variance among and within populations. These analyses were carried out with both distance indexes (*F*_*ST*_ and *R*_*ST*_), and their significance was tested with 1,000 permutations. Corrections of the significance level for multiple tests were performed following the Bonferroni procedure (Rice, 1989). The frequency of private alleles was obtained manually, observing the distribution of alleles in each locus for two analyzed populations.

Bayesian clustering was also used to assess population relatedness by means of the STRUCTURE 2.2 (Pritchard *et al.*, 2000) program. The number of populations (*K*) was estimated by using the ‘no admixture' ancestral model with correlated alleles, thereby allowing for maximal population resolution, with *K* ranging from 1 to 6. Three independent runs of 500,000 Markov chain Monte Carlo (MCMC) generations and 100,000 generations of ‘burn-in' were used for each value of *K*. The true number of populations is expected to be the value of *K* that maximizes the estimated model log-likelihood, log (P(X|*K*)) (Falush *et al.*, 2003).

The number of alleles per polymorphic locus ranged from two (*Pcor10, Pcor21, Pcor23* and *Pcor28*) to 11 (*Pcor08*), and allelic richness from 1.722 (*Pcor28*) to 9.503 (*Pcor08*) (Table 1). Nei gene diversity ranged from 0.056 (*Pcor28*) to 0.870 (P*cor01*). A total of 51 alleles were detected, of which 26 were private (Table 2). The private alleles showed frequencies ranging from 0.0167 (six alleles) to 0.3333 (one allele) (Table 2). Expected and observed heterozygosities ranged from 0.0556 (*Pcor28* - PRJ) to 0.8615 (*Pcor01 -* PRJ) and from 0.0556 (*Pcor28 -* PRJ) to 0.5556 (*Pcor08 -* PRJ and PRP), respectively (Table 1). Significant departures from HWE (p < 0.025 adjusted according to Bonferroni correction) were detected at the population level for seven loci (Table 1). The occurrence of genotyping errors due to null alleles, stuttering or large allele drop-out were checked with the MICRO-CHECKER program. Significant values were, however, not found due to stuttering or large allele drop-out. Estimates of the occurrence of null alleles revealed positive values for all cases in which departure from HWE was identified. The *F*_*IS*_ index suggested the existence of heterozygote deficiency in eight out of 14 comparisons in the populations analyzed (Table 1).

Genetic differentiation between the two populations of *P. reticulatum*, estimated through the *F*_*ST*_ index for all the loci, was 0.2290 and was statistically highly significant (p < 0.025, after Bonferroni correction), thereby showing the existence of strong genetic differentiation among the analyzed samples. The *R*_*ST*_ index estimated for all the loci was 0.1067, which was also statistically highly significant (p < 0.025, after Bonferroni correction).

Individual multi-locus genotypes were used to assign individuals to their respective population of origin. On considering the correct assignment of all individuals, 98.3% were correctly assigned to the PRP population and 99.1% to the PRJ population.

Hierarchical AMOVA revealed that most total genetic variance was to be found within populations *F*_ST_ = 77.10% and *R*_ST_ = 89.33%. The values for variability between populations were *F*_ST_ = 22.90% and *R*_ST_ = 10.67%. These were highly significant (p < 0.0001), thus revealing the strong structure of *P. reticulatum* populations.

Structure analysis without admixture inferred that the two populations were genetically distinct, with *K* = 2 populations maximizing the estimated log likelihood in the model (Figures 1  and 2).

The value of gene flow parameter *Nm* was calculated from the mean *F*_*ST*_ value. The mean value obtained was *Nm* = 0.8417, indicating that some gene exchange had occurred among the sampled populations.

The microsatellites displayed a high degree of polymorphism (mean = 7.28), consistent with the mean number observed in other fish species (DeWoody and Avise, 2000). However, Pereira *et al.* (2009) found a mean number of 15.28 alleles in six populations of *P. corruscans*.

A significant deviation of HWE (p < 0.05) was observed in seven loci within two samples analyzed (Table 1). HWE departure is, however, common with microsatellites (Alam and Islam, 2005; Carreras-Carbonell *et al.*, 2006; Chevolot *et al.*, 2006). Five potential causes may induce such a phenomenon in a given population: (i) the high number of alleles per locus, (ii) technical artifacts, such as the occurrence of null alleles, stuttering or large allele drop-out, (iii) the Wahlund effect, (iv) the selection of specific alleles, and (v) inbreeding (Hoarau *et al.*, 2002; Pereira *et al.*, 2009).

Estimates of the occurrence of null alleles revealed positive values for all cases in which HWE departure was identified (Table 1). The occurrence of null alleles is a common problem in the study of microsatellites, and may be explained by the low efficiency of the primer hybridization used to amplify some loci, due to point mutation in one or more annealing sites of these primers (Callen *et al.*, 1993; OConnell and Wright, 1997; Dakin and Avise, 2004), besides the possible differential amplification of alleles with different sizes (Wattier *et al.*, 1998). The inbreeding coefficient *F*_*IS*_ was calculated for all loci. The results acquired displayed significant departure from HWE (Table 1), thereby showing heterozygote shortage. These data show that inbreeding could be one possible cause for HWE departure. However, the cases that revealed HWE departure were those in which loci showed the largest number of alleles (Table 1). We therefore believe that the null allele hypothesis is that which best explains HWE departure.

There was significant populational structuring between the two samples of *P. reticulatum* analyzed, as demonstrated through the different tests employed in this study. The *F*_*ST*_ and *R*_*ST*_ index observed between populations of *P. reticulatum* showed high (0.2290) and moderate (0.1067) values, respectively (p < 0.025), thus suggesting there is a strong genetic structure. The differences of values between indices were probably due to differences in the mutation models on which they were based. While *F*_*ST*_ is based on IAM, *R*_*ST*_ is based on SMM. The *R*_*ST*_ index may be the best for microsatellite analysis, and it is expected that *R*_ST_ values under a strict SMM pattern would be higher than those of *F*_*ST*_ (Slatkin, 1995). However, as can be seen from our results, *F*_*ST*_ values were higher than *R*_ST_. This may be explained by the fact that probably not all microsatellite loci evolve strictly in accordance to a SMM model (Balloux and Lugon-Moulin, 2002). This presupposes that mutations occur by the addition or subtraction of a single repetition unit in the microsatellite immediately anterior or posterior to a known and highly related mutation. Departure from this strict SMM pattern would result in the inferior performance of *R*_ST_ in relation to *F*_*ST*_ (Slatkin, 1995, Balloux *et al.*, 2000). Departure from the SMM model observed in the present study may be related to the fact that in three of our eight loci repetitions were imperfect (*Pcor01* -(TC)_9_GC(TC)_9_-; *Pcor05* -(TC)_8_CC(TC)_15_- and *Pcor10* -(GTCG)_15_(GT)_9_(CC-).

The results obtained through AMOVA analysis revealed the occurrence of significant differentiation between populations of *P. reticulatum* on using both indices (*F*_*ST*_ and *R*_*ST*_), thereby depicting the occurrence of strong genetic structuring. The values of molecular variation among populations were 22.90% (*F*_*ST*_) and 10.67% (*R*_*ST*_), both with highly significant *p* values (p < 0.0001). Through structure analysis without admixture, it was shown that both populations were genetically distinct, with *K* = 2 populations maximizing the estimated log-likelihood in the model (Figures 1  and 2).

The genetic flow of all loci in the two populations was estimated as *Nm =* 0.8417 migrants per generation. According to Nei (1987), *Nm* values above 1 suggest that genetic flow constitutes a positive factor against genetic differentiation among populations (Spieth, 1974). Thus, our data showed that genetic flow between the two populations analyzed did not exist or was very low, thereby reinforcing the hypothesis of genetic structure. The results obtained in the assignment tests were extremely positive, as described in the literature for other fish groups (Triantafyllidis *et al.*, 2002). In the present study involving seven microsatellites (*F*_*ST*_ = 0.2290), 98.3% and 99.1% of the individuals were correctly assigned to the location from which they were sampled (PRP and PRJ, respectively). These results are consistent with the values found by Pereira *et al.* (2009) when analyzing six *P. corruscans* populations (values ranging from 93.6% to 98.2%). Cornuet *et al.* (1999) showed by simulations that 100% correct assignments can be achieved through the Bayesian method with as few as 10 microsatellite loci and 10 individuals sampled per population, when populations are sufficiently diverged (*F*_*ST*_ ~ 0.1).

**Figure 1:**
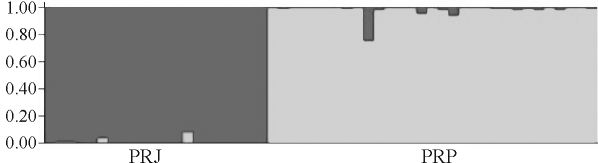
Structure bar-plot representing assignments of genotypes to each population. Grays represent assignments assuming two populations, demonstrating the pattern of clustering within regional groups. PRJ = *Pseudoplatystoma reticulatum* Jauru River; PRP = *P. reticulatum* Paraguai River.

**Figure 2:**
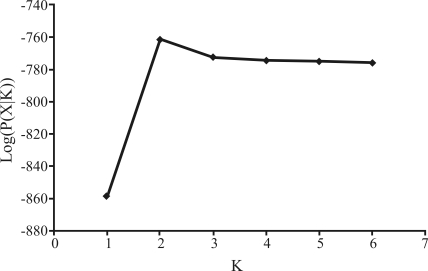
Results of the Structure 2.2 analysis of estimated log likelihood log (P(X|*K*)) for the model *vs.* the number of populations, *K* to populations of *P. reticulatum*. Points are averages of three independent runs of 100,000 generations of ‘burn-in' followed by 500,000 MCMC generations.

Twenty-six private alleles were found in the two populations of *P. reticulatum* analyzed. In five of these, frequency was higher than 10% (Table 2). The existence of private alleles places in evidence the absence of gene flow, or at least that it is at a minimum and frequently present in structured populations. Thus, these data reinforce the existence of firm structuring in the *P. reticulatum* analyzed. Furthermore, all the tests employed confirmed the strong structure in these populations. Thus, our work renders preliminary evidence of the genetic structure in *P. reticulatum*. As the results presented are consistent with those obtained for *P. corruscans* (Pereira *et al.*, 2009) and, on considering the similar behavior between these two species, we suggest that *P. reticulatum* also presents homing behavior.

The ability to identify and define biological populations is crucial for taking informed decisions concerning conservation and management (Waples and Gaggiotti, 2006). Considering the ecological and economic importance of *P. reticulatum*, the present data constitute a particularly important element when contemplating their management and conservation. It also places in evidence, the importance of preserving each population to further a positive outcome in the genetic conservation of these species.

## Figures and Tables

**Table 1:** Summary of microsatellite data on each analyzed population of *Pseudoplatystoma reticulatum* analyzed. *N*, Number of individuals; *A*, number of alleles; AR, Allelic Richness; ND, Nei gene diversity; *H*_O_, observed heterozygosity; *H*_E_, expected heterozygosity; *F*_IS_, inbreeding coefficient; HWE result of Hardy-Weinberg probability test on deviation from expected Hardy-Weinberg proportions with *p*-*value* = 0.05 (adjustment Bonferroni correction p = 0.025; *k* = 2), *, significant; ns, not significantand *r*, null allele frequency per loci.

Loci	*Pcor01*	*Pcor05*	*Pcor08*	*Pcor10*	*Pcor21*	*Pcor23*	*Pcor28*
*P. reticulatum* Jauru (PRJ)							
*N*	20	21	18	13	21	21	18
*A*	9	8	11	2	4	2	2
AR	8.132	7.085	9.503	2.000	3.475	2.000	1.722
ND	0.870	0.813	0.856	0.269	0.337	0.343	0.056
*Ho*	0.5500	0.3333	0.5556	0.3077	0.2857	0.3333	0.0556
*He*	0.8615	0.8014	0.8508	0.2708	0.3357	0.2846	0.0556
*Fis*	0.3452	0.5739	0.3284	-0.1818	0.1280	-0.2000	-0.0286
HWE	(0.0010)*	(0.0000)*	(0.0000)*	(1.0000)ns	(0.0738)ns	(0.5324)ns	(-) ns
*r*	0.1576	0.2519	0.1472	-	-	-	-

*P. reticulatum* Paraguai (PRP)							
*N*	27	31	18	31	25	30	30
*A*	5	10	8	3	2	7	5
AR	4.888	7.430	8.824	2.706	1.775	5.597	3.641
ND	0.661	0.756	0.819	0.210	0.078	0.767	0.301
*Ho*	0.3704	0.5312	0.5556	0.2258	0.0800	0.2667	0.3000
*He*	0.6401	0.7361	0.7921	0.2089	0.0784	0.7605	0.3006
*Fis*	0.4105	0.2668	0.2786	-0.0987	-0.0417	0.6434	-0.0150
HWE	(0.0038)*	(0.0000)*	(0.0024)*	(1.0000)ns	(1.0000)ns	(0.0000)*	(0.2382)ns
*r*	0.1439	0.1185	0.1183	-	-	0.2557	-

**Table 2:** Private allele counts. Allele number and relative frequency (in parentheses) are listed for each locus analyzed.

	*Pcor01*	*Pcor05*	*Pcor08*	*Pcor10*	*Pcor21*	*Pcor23*	*Pcor28*
Jauru	107 (0.1500)	133 (0.0476)	165 (0.0278)		104 (0.0238)		
(PRJ)	109 (0.1250)		179 (0.0278)		114 (0.1190)		
	119 (0.0250)				120 (0.0476)		
	121 (0.0500)						
Paraguai	151 (0.0185)	137 (0.0167)	147 (0.0263)	139 (0.0484)	116 (0.0400)	091 (0.3333)	100 (0.0167)
(PRP)		145 (0.0167)				095 (0.2333)	102 (0.0500)
		153 (0.0333)				097 (0.0500)	106 (0.0167)
		159 (0.0167)				101 (0.0167)	
						107 (0.0167)	

## References

[AlamandIslam2005] Alam M.S., Islam M.S. (2005). Population genetic structure of *Catla catla* (Hamilton) revealed by microsatellite DNA markers. Aquaculture.

[AllanandFlecker1993] Allan J.D., Flecker A.S. (1993). Biodiversity conservation in running waters: Identifying the major factors that threaten destruction of riverine species and ecosystems. BioSciences.

[BallouxandLugon-Moulin2002] Balloux F., Lugon-Moulin N. (2002). The estimation of population differentiation with microsatellite markers. Mol Ecol.

[Ballouxetal2000] Balloux F., Brünner H., Lugon-Moulin N., Hausser J., Goudet J. (2000). Microsatellites can be misleading: An empirical and simulation study. Evolution.

[Barrosoetal2005] Barroso R.M., Hilsdorf A.W.S., Moreira H.L.M., Cabello P.H., Traub-Cseko Y.M. (2005). Genetic diversity of wild and cultured populations of *Brycon opalinus* (Cuvier, 1819) (Characiformes, Characidae, Bryconiae) using microsatellites. Aquaculture.

[BarthemandGoulding2007] Barthem R., Goulding M. (2007). Um Ecossistema Inesperado: A Amazônia Revelada Pela Pesca.

[Callenetal1993] Callen D.F., Thompson A.D., Shen Y., Phillipis H.A., Richards R.I., Mulley J.C., Sutherland G.R. (1993). Incidence and origin of `null' alleles in the (AC)_*n*_ microsatellite markers. Am J Hum Genet.

[Carreras-Carbonelletal2006] Carreras-Carbonell J., Macpherson E., Pascual M. (2006). Population structure within and between subspecies of the Mediterranean triplefin fish *Tripterygion delaisi* revealed by highly polymorphic microsatellite loci. Mol Ecol.

[Catella2003] Catella A.C. (2003). A Pesca no Pantanal Sul: Situação Atual e Perspectivas.

[Chevolotetal2006] Chevolot M., Ellis J.R., Hoarau G., Rijnsdorp A.D., Stam W.T., Olsen J.L. (2006). Population structure of the thornback ray (*Raja clavata* L. ) in British waters. J Sea Res.

[Cornuetetal1999] Cornuet J.M., Piry S., Luikart G., Estoup A., Solignac M. (1999). New methods employing multilocus genotypes to select or exclude populations as origins of individuals. Genetics.

[DakinandAvise2004] Dakin E.E., Avise J.C. (2004). Microsatellite null alleles in parentage analysis. Heredity.

[DeWoodyandAvise2000] DeWoody J.A., Avise J.C. (2000). Microsatellite variation in marine, freshwater and anadromous fishes compared with other animals. J Fish Biol.

[EhrlichandEhrlich1992] Ehrlich P., Ehrlich A. (1992). The value of biodiversity. Ambio.

[Excoffieretal1992] Excoffier L., Smouse P., Quattro J. (1992). Analysis of molecular variance inferred from metric distances among DNA haplotypes: Application to human mitochondrial DNA restriction data. Genetics.

[Excoffieretal2005] Excoffier L., Laval G., Schneider S. (2005). Arlequin v. 3.0: An integrated software package for population genetics data analysis. Evol Bioinform Online.

[Falushetal2003] Falush D., Stephens M., Pritchard J.K. (2003). Inference of population structure using multilocus genotype data: Linked loci and correlated allele frequencies. Genetics.

[Ferraris2007] Ferraris C.J. (2007). Checklist of catfishes, recent and fossil (Osteichthyes, Siluriformes), and catalogue of siluriform primary types. Zootaxa.

[Hoarauetal2002] Hoarau G., Rijnsdorp A.D., Van der Veer H.W., Stam W.T., Olsen J.L. (2002). Population structure of plaice (*Pleuronectes platessa* 1) in northern Europe: Microsatellites revealed large-scale spatial and temporal homogeneity. Mol Ecol.

[Koskinenetal2002] Koskinen M.T., Nilsson J., Veselov A.J., Potutkin A.G., Ranta E., Primmer C.R. (2002). Microsatellite data resolve phylogeographic patterns in European grayling, *Thymallus thymallus*, Salmonidae. Heredity.

[Makinenetal2006] Mäkinen H.S., Cano J.M., Meriläl J. (2006). Genetic relationships among marine and freshwater populations of the European three-spined stickleback (*Gasterosteus aculeatus*) revealed by microsatellites. Mol Ecol.

[Miranda1997] Miranda M.O.T. (1997). Surubim.

[MoyleandLeidy1992] Moyle P.B., Leidy R.A., Fielder P.L., Jain S.K. (1992). Loss of biodiversity in aquatic ecosystems: Evidence from fish faunas. Conservation Biology: The Theory and Practice of Nature Conservation, Preservation, and Management.

[Nei1987] Nei M. (1987). Molecular Evolutionary Genetics.

[OConnellandWright1997] O'Connell M., Wright J.M. (1997). Microsatellite DNA in fishes. Rev Fish Biol Fish.

[Pereiraetal2009] Pereira L.H.G., Foresti F., Oliveria C. (2009). Genetic structure of the migratory catfish *Pseudoplatystoma corruscans* (Siluriformes, Pimelodidae) suggests homing behavior. Ecol Freshw Fish.

[Pritchardetal2000] Pritchard J.K., Stephens M., Donnelly P. (2000). Inference of population structure using multilocus genotype data. Genetics.

[RaymondandRousset1995] Raymond M., Rousset F. (1995). Genepop v. 1.2: Population genetics software for exact tests and ecumenicism. J Hered.

[Resende2003] Resende E.K., Carolsfeld J., Harvey B., Ross C., Baer A. (2003). Migratory fishes of the Paraguay - Paraná Basin excluding the Upper Paraná Basin. Migratory Fishes of South America. Biology, Fisheries and Conservation Status.

[Revaldavesetal2005] Revaldaves E., Pereira L.H.G., Foresti F., Oliveira C. (2005). Isolation and characterization of microsatellite loci in *Pseudoplatystoma corruscans* (Siluriformes, Pimelodidae) and cross-species amplification. Mol Ecol Notes.

[Rice1989] Rice W.R. (1989). Analyzing tables of statistical tests. Evolution.

[Salgueiroetal2003] Salgueiro P., Carvalho G., Collares-Pereira M.J., Coelho M.M. (2003). Microsatellite analysis of genetic population structure of the endangered cyprinid *Anaecypris hispanica* in Portugal: Implications for conservation. Biol Conserv.

[Satoetal1988] Sato Y., Cardoso E.L., Sallum W.B. (1988). Reprodução induzida do surubim (*Pseudoplatystoma coruscans*) da bacia do rio São Francisco. Encontro Anual de Aqüicultura - Associação Mineira de Aqüicultura, Belo Horizonte, pp.

[Slatkin1995] Slatkin M. (1995). A measure of population subdivision based on microsatellite allele frequencies. Genetics.

[Spieth1974] Spieth P.T. (1974). Gene flow and genetic differentiation. Genetics.

[Triantafyllidisetal2002] Triantafyllidis A., Krieg F., Cottin C., Abatzopoulos T.J., Triantafyllidis C., Guyomard R. (2002). Genetic structure and phylogeography of European catfish (*Silurus glandis*) populations. Mol Ecol.

[vanOosterhoutetal2004] van Oosterhout C., Hutchinson W.F., Wills D.P.M., Shipley P. (2004). Micro-Checker: Software for identifying and correcting genotyping errors in microsatellite data. Mol Ecol Notes.

[ViladaSilva1995] Vila da Silva J.S., Esteves F.A. (1995). Elementos fisiográficos para delimitação do ecossistema Pantanal: Discussão e proposta. Oecologia Brasiliensis, Estrutura, Funcionamento e Manejo de Ecossistemas Brasileiros.

[WaplesandGaggiotti2006] Waples R.S., Gaggiotti O. (2006). What is a population? An empirical evaluation of some genetic methods for identifying the number of gene pools and their degree of connectivity. Mol Ecol.

[Wattieretal1998] Wattier R., Engel C.R., Saumitou-Laprade P., Valero M. (1998). Short allele dominance as a source of heterozygote deficiency at microsatellite loci: Experimental evidence at the dinucleotide locus Gv1CT in *Gracilaria gracilis* (Rhodophyta). Mol Ecol.

[WeirandCockerham1984] Weir B.S., Cockerham C.C. (1984). Estimating *F*-statistics for the analysis of population structure. Evolution.

[WrightandBentzen1994] Wright J.M., Bentzen P., Carvalho G.R., Pitcher T.J. (1994). Microsatellites: Genetic markers for the future. Molecular Genetics in Fisheries.

[YehandBoyle1997] Yeh F.C., Boyle T.J.B. (1997). Population genetic analysis of co-dominant and dominant markers and quantitative traits. Belg J Bot.

